# The Impact of Lymphatic Filariasis Mass Drug Administration Scaling Down on Soil-Transmitted Helminth Control in School-Age Children. Present Situation and Expected Impact from 2016 to 2020

**DOI:** 10.1371/journal.pntd.0005202

**Published:** 2016-12-19

**Authors:** Denise Mupfasoni, Antonio Montresor, Alexei Mikhailov, Jonathan King

**Affiliations:** Department of Control of Neglected Tropical Diseases, World Health Organization, Geneva, Switzerland; Centers for Disease Control and Prevention, UNITED STATES

## Abstract

Lymphatic filariasis (LF) and soil-transmitted-helminths (STH) are co-endemic in 58 countries which are mostly in Africa and Asia. Worldwide, 486 million school-age children are considered at risk of both diseases. In 2000, the World Health Organization (WHO) established the global programme to eliminate LF by 2020. Since then, the LF elimination programme has distributed ivermectin or diethylcarbamazine citrate (DEC) in combination with albendazole, thereby also treating STH. Consequently, many school-age children have been treated for STH through the LF programme. As treatment targets towards the 2020 LF elimination goal are achieved, many countries are implementing the transmission assessment survey (TAS) and, if the LF prevalence is estimated to be less than 1%, scaling down mass drug administration (MDA). We analysed the 2014 data on preventive chemotherapy (PC) reported from LF STH co-endemic countries and projected the year and location of TAS expected to be conducted between 2016 and 2020 to assess the impact of this scaling down on STH PC. Eighty percent of all co-endemic countries that have already stopped LF MDA nationally were able to establish STH PC through schools. It is estimated that 14% of the total number of children presently covered by the LF programme is at risk of not continuing to receive PC for STH. In order to achieve and maintain the WHO 2020 goal for STH control, there is an urgent need to establish and reinforce school-based deworming programmes in countries scaling-down national LF elimination programmes.

## Author Summary

Lymphatic filariasis (LF) and soil-transmitted helminths (STH) (i.e. intestinal worms) are two tropical diseases that are found together in 58 countries in the world. School-age children are most affected by intestinal worms, albendazole, one of the two drugs used for LF, also treats STH. For this reason, large-scale delivery of LF drugs in the community has been used as a means to also treat school-age children for intestinal worm infections in many countries. In line with the WHO goal to eliminate LF by 2020, countries that have achieved that objective have started stopping community-based LF treatment. Therefore, we analysed treatment data from 2014 to quantify the effect of this reduction on treatment of school-age children for intestinal worms. The results show that 80% of countries that have already stopped LF treatment were able to administer deworming drugs for STH to school-age children within school-based treatment programmes. There is an urgent need to continue to establish and strengthen deworming through school health programmes in endemic countries in order to meet the WHO established goal to treat at least 75% of at risk school-age children for STH by 2020.

## Introduction

**Fig 1:**
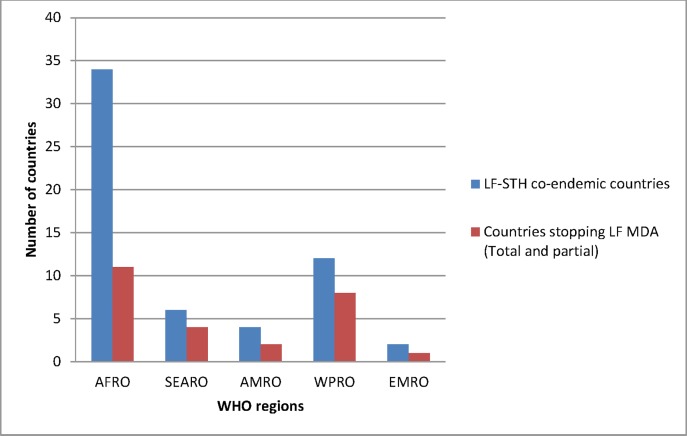
Number of LF and STH co-endemic countries within WHO regions.

**Fig 2:**
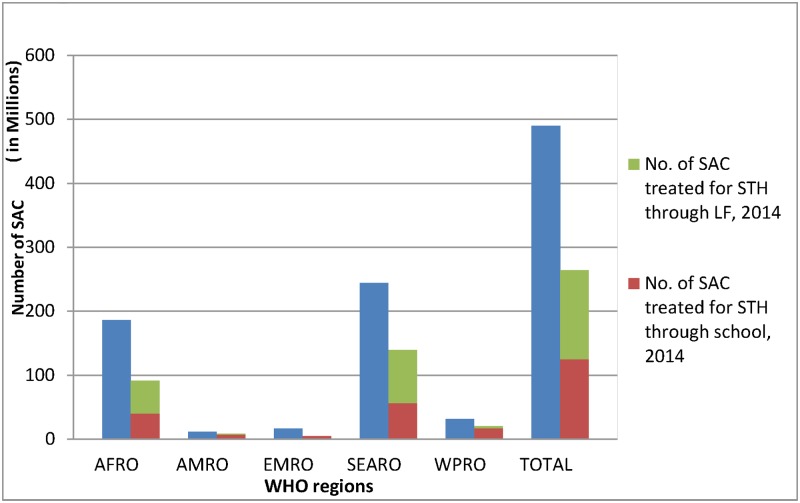
Number of school-age children dewormed for STH in LF-endemic countries in 2014.

Lymphatic filariasis (LF) and soil-transmitted helminth (STH) infections are co-endemic in 58 countries worldwide: 34 in Africa region, 12 in Western Pacific region, six in South-East Asia region, four in America region and two in Eastern Mediterranean region (Fig 1). Mass drug administration (MDA) is the recommended preventive chemotherapy (PC) strategy of delivering a combination of albendazole and ivermectin or diethylcarbamazine citrate (DEC) for LF elimination. Since albendazole also treats STH infections [1], MDA for LF has often replaced specific interventions targeting STH in co-endemic areas. In 2014, it was estimated that 46% of those school-age children (SAC) who received PC for STH received anthelminthics through MDA targeting LF while 54% were dewormed through school-based interventions (Fig 2).

LF is targeted for global elimination as a public health problem by 2020 [2]. At least 5 years of MDA with effective coverage is expected to have reduced LF infection to a level at which transmission can no longer be sustained and thus MDA can stop. WHO recommends the transmission assessment survey (TAS) as the decision-making tool to determine when to stop MDA [3]. Where the number of LF infected children is less than or equal to the critical cut-off value the assessment is considered ‘passed’ and MDA may cease. After stopping MDA, countries are expected to conduct TAS twice over a 4–5 year period to confirm whether LF elimination has been achieved and sustained [4].

On the other hand, for STH, the WHO target for 2020 is to treat at least 75% of SAC living in STH endemic countries [5]. Using a school-based platform to distribute anthelminthic treatment is one of the most cost-effective approaches that WHO recommends in STH control strategies.

The aim of this study is to assess the effect of the progressive scaling down of MDA for LF on STH control and the status of the measures in place by different countries to mitigate this effect.

## Materials and Methods

LF and STH are two neglected tropical diseases (NTDs) that put SAC at great risk of infection and morbidity. Depending on the STH endemicity of a country, some SAC are treated once a year and others twice a year, while, for LF, the treatment is annually and community-wide.

WHO established the preventive chemotherapy and transmission control (PCT) databank in order to facilitate information access and sharing among national programmes of endemic countries and NTD partners [6]. Each year the database is updated with data on MDA reported by endemic countries [7]. The tools used to collect data and the methodology utilized to summarize it have been described elsewhere [8].

From the 2014 PCT databank we calculated the following numbers:

- number of implementation units (IUs) that conducted TAS by 2014 and passed (by country)
- number of SAC living in IUs that passed TAS
- number of SAC living in IUs that passed TAS that were treated for STH by a school programme

From internal WHO files on TAS we calculated:

- the number of IUs where TAS is expected to be conducted between 2015 and 2020
- the number of SAC living in IUs where TAS is expected to be conducted (assuming all IUs conducting TAS will pass)
- the total number of SAC living in IUs where a school intervention for STH control is already in place in 2014 or 2015

### Ethics statement

The study is a secondary analysis of aggregated data officially reported annually by Ministries of Health of endemic countries to WHO. The data are publicly available and anonymous.

## Results

### AFRICA Region (AFR)

In 2014, of the 34 co-endemic countries for LF and STH in Africa region, LF MDA was implemented at least partially in 20 countries. Six countries (Benin, Cameroon, Cote d’Ivoire, Madagascar, Mozambique and Senegal) reached at least 75% of STH treatment coverage. More than half of SAC in need of STH PC were treated through LF MDA or PC with schools as drug distribution channels (Table 1). Two countries, Togo and Malawi, stopped LF MDA nationwide after they implemented and passed the first TAS (i.e. TAS1),STH PC for school-age children was conducted in both countries (Table 2) but Malawi was not able to reach the 100% geographic coverage. Other countries like Benin, Burkina Faso, Ghana, Mali, Nigeria, and Tanzania stopped LF MDA in 87 IUs (Table in S1 Table) and the data analysis in those IUs demonstrated that STH PC for school-age children was conducted in all countries except in Burkina Faso and Mali. Sierra Leone, Guinea and Liberia did not implement LF MDA in 2014 due to the Ebola virus epidemic.

**Table 1:** Summary table of number of SAC treated for STH in LF-endemic countries in WHO regions, 2014.

		LF non- endemic		LF endemic		PC stopped		LF endemicity unknown		Total	
	Indicator	# IU	SAC	# IU	SAC	# IU	SAC	# IU	SAC	# IU	SAC*
**AFRO**	TOTAL	1,704	81,054,011	1,818	95,881,982	87	4,812,971	58	2,458,040	3,667	184,207,004
	Treated LF	4	109,952	1,129	49,803,435	5	212,116	0	0	1,138	50,125,503
	Treated STH	521	19,680,559	599	21,431,675	39	1,950,855	3	2,775	1,162	43,065,864
	Overlapping	2	55,207	435	12,677,897	2	81,378	0	0	439	12,814,482
**AMRO**	TOTAL	22	1,574,968	123	2,723,988	66	9,121,568	0	0	211	13,420,524
	Treated LF	0	0	81	1,325,787	0	0	0	0	79	1,325,787
	Treated STH	22	1,172,786	11	759,163	32	4,934,866	0	0	65	6,866,815
	Overlapping	0	0	3	103,567	0	0	0	0	3	103,567
**EMRO**	TOTAL	24	1,279,742	30	1,694,741	0	0	130	8,126,024	184	11,100,507
	Treated LF	0	0	0	0	0	0	0	0	0	0
	Treated STH	2	5,533	0	0	0	0	12	325,493	14	331,026
	Overlapping	0	0	0	0	0	0	0	0	0	0
**SEARO**	TOTAL	338	44,333,448	485	129,052,478	141	45,171,143	1	326,310	965	218,883,379
	Treated LF	0	0	336	82,163,334	0	0	0	0	336	82,163,334
	Treated STH	69	18,439,645	116	23,252,269	43	7,465,774	0	0	228	49,157,688
	Overlapping	0	0	98	14,006,083	0	0	0	0	98	14,006,083
**WPRO**	TOTAL	36	11,142,460	22	4,218,431	23	3,894,861	0	0	81	19,255,752
	Treated LF	0	0	22	4,218,431	0	0	0	0	22	4,218,431
	Treated STH	27	4,571,347	17	1,980,755	18	1,579,257	0	0	62	8,131,359
	Overlapping	0	0	17	1,909,761	0	0	0	0	17	1,909,761

*SAC population includes districts with STH prevalence below 20%

(Source of data: PCT databank, http://www.who.int/neglected_diseases/preventive_chemotherapy/databank/en/)

**Table 2:** STH preventive chemotherapy status in countries that have completely stopped LF MDA.

Countries	# SAC in need of STH PC	# SAC treated through LF MDA (before stopping MDA)	# SAC treated through schools (2014 data)
Malawi	4,703,907	4,194,811	1,997,024
Togo	1,993,687	252,949	1,055,597
Yemen	6,395,950	11,521	4,910,901
Cambodia	4,194,270	106,397	3,987,461
Vietnam	4,580,664	0	3,586,660
Kiribati	23,293	1,618	13,169
Marshall Islands	13,510	126	5,143
Tonga	26,075	0	0
Vanuatu	67,299	39,690	38,392
**Total**	**26,273,937**	**4,607,112**	**15,594,347**

In the period 2016–2020, TAS1 is expected to be conducted in 1866 IUs in the 12 countries that have started LF MDA scaling down. The total number of SAC living in those areas is 73 million of which 18 million are at risk of not continuing to receive PC for STH.

### Region of America (AMR)

In this region, there are four LF STH co-endemic countries: Brazil, Dominican Republic, Guyana and Haiti. In 2014, Brazil stopped LF MDA in 27 endemic IUs and remained with 2 IUs that are still treating for LF until 2017, while the Dominican Republic stopped LF MDA in five IUs. Moreover, these two countries were able to implement STH PC for school-age children nationally while in Haiti, SAC were dewormed through both LF MDA and school intervention. In Guyana, school-age children were dewormed for STH only through LF MDA (Table in S1 Table). Two countries, Dominican Republic and Haiti surpassed WHO target for STH control by treating 100% and 91% of SAC respectively.

In the period 2016–2020, TAS1 is expected to be conducted in 134 IUs in four countries. The total number of SAC living in those areas is 3 million of which 900,000 are presently not covered by a school programme targeting STH.

### South-East Asia Region (SEAR)

**Fig 3:**
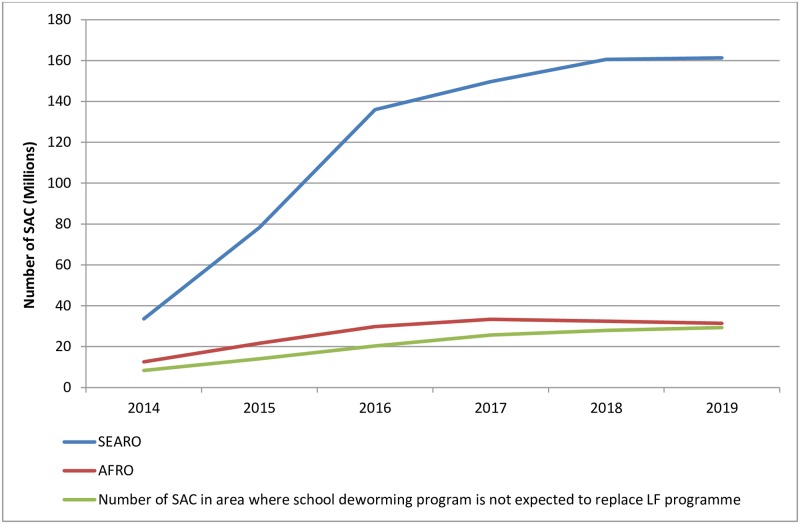
Number of at risk SAC in areas that have stopped or plan to stop LF MDA by 2020.

Among the nine LF-endemic countries, six are co-endemic with STH infections: Bangladesh, India, Indonesia, Myanmar, Nepal and Timor-Leste. All of these except Timor-Leste have implemented TAS and stopped MDA in some IUs. The most significant scale-down has occurred in India and Bangladesh with 71 and 18 IUs having already stopped LF MDA, respectively. Looking at the 2014 STH PC data in those IUs, school-aged children were dewormed for STH in Bangladesh and in one state in India. Nepal and Myanmar conducted LF MDA in endemic areas and dewormed school-age children for STH nationally. Both Bangladesh and Myanmar achieved the 75% minimum treatment coverage in this age group. Indonesia reported deworming of SAC for STH only in LF-endemic areas while Timor-Leste did not conduct any MDA in 2014 (Table in S1 Table). By the end of 2016, the region will have more than 100 million SAC at risk of STH in IUs where LF MDA will stop, most of whom are from India (Fig 3). However, India launched a national deworming programme in 2015 which aims to scale up in areas where LF MDA will scale down [9].

In the period 2016–2020, TAS1 is expected to be conducted in 467 implementation units in 5 countries. The total number of SAC living in those areas is 132 million of which 9 million are presently not covered by a school programme targeting STH.

### Eastern Mediterranean Region (EMR)

Sudan and Yemen are the two countries in the region that require both LF and STH PC. Yemen is in post LF MDA surveillance while Sudan is finalising the mapping to refine the population requiring LF MDA. In 2014, Yemen dewormed SAC and achieved WHO minimum target for STH control while Sudan implemented STH PC for school-age children in 12 IUs (Table 2 and Table in S1 Table).

### Western Pacific Region (WPR)

In this region, among the 22 LF-endemic countries, 12 are co-endemic with STH, 6 of which have already stopped LF MDA nationally and are under post-MDA surveillance. Among these, only one country, Tonga, did not conduct STH PC for school-age children in 2014. Philippines, Cambodia and Vietnam have the highest number of school-age children requiring treatment and in 2014 they all conducted STH PC for school-age children (Table 2). The last two countries attained the 75% and 100% STH treatment coverage and geographic coverage targets respectively and have already stopped the LF MDA. Moreover, Philippines has scaled down LF MDA significantly: 23 IUs have already stopped LF MDA and school-age children were dewormed for STH in 18 IUs (Table in S1 Table)). Micronesia and Papua New Guinea dewormed school-age children only through the LF programme.

In the period 2016–2020, TAS1 is expected to be conducted in 17 IUs in four countries. The total number of SAC living in those areas is 6 million of which 2 million are not covered by a school programme targeting STH.

Globally, nine STH LF co-endemic countries have already stopped LF MDA nationally. Seven of them (80%) have been able to transition successfully to STH deworming for school-age children through schools and three were able to achieve and maintain the WHO minimum STH PC target. However, Malawi and Tonga were not able to reach the total number of school-age children that were routinely dewormed through LF (Table 2). Among the 41 countries that have stopped LF MDA, at least partially, five (12%) have been able to successfully transition to school deworming for STH in all IUs that stopped LF MDA, ten (24%) treated at least 75% of SAC requiring PC.

Of 15 countries that are expecting to stop LF MDA in some IUs by 2020, more than 85% have school deworming programmes which have taken on deworming of SAC in all IUs that stopped LF MDA. However, three countries (Burkina Faso, Mali and India) that have school deworming programmes were unable to deworm school-age children in all areas where LF MDA has already stopped in 2014.

From 2016 and onward, the number of SAC at risk of STH in areas stopping LF MDA will increase significantly reaching 160 million. Since the greatest proportion will be in countries that have already put in place a national STH deworming programme expecting to cover all SAC in need of treatment, the expected number of children not covered by school deworming programmes is estimated to be around 30 million globally (Fig 3).

To facilitate the identification of countries that need to consider this imminent need for scaling-up school-based deworming, we divided them into three categories: category one includes countries that have completely or partially stopped LF MDA and which were already able to successfully continue STH PC for school-age children through schools; category two includes countries that have completely or partially stopped LF MDA and which conducted STH PC for school-age children, but did not reach those that were routinely dewormed through LF and category three includes countries that do not have a national school deworming programme (Table 3).

**Table 3:** LF-endemic categorisation of countries by STH PC implementation for SAC.

Category 1: countries that have completely or partially stopped LF MDA and which were able to continue STH PC for SAC through schools	Category 2: countries that have completely or partially stopped LF MDA and which conducted STH PC for SAC, but did not reach those that were routinely dewormed through LF	Category 3: countries that have completely or partially stopped LF MDA and which do not have a nation-wide school deworming programme
Bangladesh	Benin	Indonesia
Brazil	Burkina Faso	Tonga
Cambodia	India	
Dominican Republic	Malawi	
Ghana	Mali	
Kiribati	Tanzania	
Marshall Islands		
Nigeria		
Nepal		
Togo		
Vanuatu		
Vietnam		
Yemen		

## Discussion

LF MDA has been used for many years as a platform for integrated PC to control diseases including STH and as a community-based drug distribution programme; it has reached billions of people [10] which includes SAC. The analysis of 2014 data shows that in all LF-endemic WHO regions, LF MDA has started scaling down, with nine STH co-endemic countries having already stopped MDA at the national level. School-based deworming is a safe, simple and cost-effective control strategy for STH infections recommended by WHO, that can reach both enrolled and non-enrolled school-age children [11]. With LF MDA scaling down whether these two infections co-exist, countries should effectively transition from LF MDA to school-based deworming for STH control after assessing the epidemiology of STH infection to determine whether STH PC should continue in the absence of LF MDA.

The results of 2014 data analysis demonstrate that only a minority of the total number of children presently covered by LF programmes are not currently covered by a school deworming programme. Most of these SAC children are in African countries. This is the geographical region where most attention should be focused. The results also demonstrate that some countries have been more successful than others in achieving this transition from LF MDA dependent deworming of SAC to school-based deworming programmes. Three factors contributing to this success included the establishment of a school-based deworming programme before the end of LF MDA, integration of STH data collection with TAS and intersectoral mobilisation of resources to sustain STH deworming activities.

Countries that have transitioned effectively to school- based PC are mainly the ones that had simultaneously implemented school and community based PC interventions in collaboration with other sectors, notably with the education sector (e.g. countries in category 1, Table 3). Case studies from World Bank [12] describe Cambodia and Vietnam as examples of countries that have achieved and sustained WHO target of treating at least 75% of SAC, attributing their success to multisectoral collaboration. Consequently, WHO/NTD is working with multiple partners (e.g. World Bank, Global Partnership for Education, NGOs) to forge collaboration between health and education sectors at all levels. Such collaborations facilitate the mobilization of resources required to support school-based deworming programmes. In the meantime, some countries are establishing national school-based deworming programmes on their own initiative. For example India launched a national school-based deworming programme in 2015 that is expected to cover all SAC in the country, a total of 136 million, which is 24% of SAC population in need of STH PC worldwide.

Impact studies on the epidemiology of STH during LF TAS allow national programme managers to assess the prevalence and intensity of infection in the target population and thereby adjust the frequency of deworming interventions in the school-age population. WHO has published a protocol to help countries integrate STH data collection in TAS [13]. Two studies conducted in Sri Lanka and Burkina Faso on assessment of STH morbidity and prevalence during LF TAS enabled these countries to review their STH control strategies leading up to 2020 [14, 15]. STH data collected during TAS showed that in Burkina Faso SAC do not need deworming in area where LF MDA stopped; the STH prevalence and intensity had dropped significantly and the country is planning to review its strategy to consolidate these gains. Additionally, WHO urges partners that were supporting LF MDA to continue to support countries in their transition to conduct school-based deworming for SAC and STH epidemiological surveys during LF TAS as countries work to establish sustainable STH control strategies post LF MDA.

In conclusion, the scaling down of LF MDA will affect STH PC. Many countries have school based deworming programmes that will take over and sustain the gains acquired during the LF elimination programme. WHO urges countries to undertake STH epidemiological surveys along with LF TAS which will allow them to determine their appropriate strategy for the control of STH in SAC with an emphasis on integration into existing government structures and multisectoral collaboration. Additionally, future analysis should examine the public health effects of LF MDA deworming on women of childbearing age and similarly review the implications of the ending of LF MDA for this important age-group also at risk of STH infection and its associated complications.

## Supporting Information

S1 Table Detailed table of number of SAC treated for STH in LF-endemic countries in WHO regions, 2014. (DOCX)
