# Supplementary material for: The Impact of Lymphatic Filariasis Mass Drug Administration Scaling Down on Soil-Transmitted Helminth Control in School-Age Children. Present Situation and Expected Impact from 2016 to 2020
Source: PLoS Negl Trop Dis. 2016 Dec 19;10(12):e0005202. doi: 10.1371/journal.pntd.0005202 (PMC5167227; doi:10.1371/journal.pntd.0005202)
Supplement: S1 Table — (DOCX) [file pntd.0005202.s001.docx]

**Supporting information files**

**Table 1. Detailed table of number of SAC treated for STH in LF-endemic countries in WHO regions, 2014**

|  |  | **No LF endemic** | | **LF Endemic** | | **LF MDA stopped** | | **LF endemicity Unknown** | | **Total** | |
| --- | --- | --- | --- | --- | --- | --- | --- | --- | --- | --- | --- |
| **COUNTRY** | **INDICATORS** | **# IU** | **SAC** | **# IU** | **SAC** | **# IU** | **SAC** | **# IU** | **SAC** | **# IU** | **SAC** |
| **AFRO** |  |  |  |  |  |  |  |  |  |  |  |
| BENIN | TOTAL | 14 | 474,941 | 1 | 73,683 | 23 | 717,104 | 0 | 0 | 38 | 1,265,728 |
|  | Treated LF | 0 | 0 | 1 | 22,434 | 0 | 0 | 0 | 0 | 1 | 22,434 |
|  | Treated STH | 10 | 197,163 | 1 | 45,789 | 6 | 115,266 | 0 | 0 | 17 | 358,218 |
|  | Overlapping | 0 | 0 | 1 | 22,434 | 0 | 0 | 0 | 0 | 1 | 22,434 |
|  | Gap | 4 | 277,778 | 0 | 5,460 | 17 | 601,838 | 0 | 0 | 21 | 885,076 |
| BURKINA FASO | TOTAL | 0 | 0 | 41 | 3,415,905 | 22 | 1,808,526 | 0 | 0 | 63 | 5,224,431 |
|  | Treated LF | 0 | 0 | 41 | 2,618,949 | 0 | 0 | 0 | 0 | 41 | 2,618,949 |
|  | Treated STH | 0 | 0 | 0 | 0 | 0 | 0 | 0 | 0 | 0 | 0 |
|  | Overlapping | 0 | 0 | 0 | 0 | 0 | 0 | 0 | 0 | 0 | 0 |
|  | Gap | 0 | 0 | 0 | 796,956 | 22 | 1,808,526 | 0 | 0 | 22 | 2,605,482 |
| CAMEROON | TOTAL | 30 | 1,459,175 | 159 | 4,271,679 | 0 | 0 | 0 | 0 | 189 | 5,730,854 |
|  | Treated LF | 0 | 0 | 132 | 4,171,161 | 0 | 0 | 0 | 0 | 132 | 4,171,161 |
|  | Treated STH | 25 | 1,105,065 | 157 | 3,913,256 | 0 | 0 | 0 | 0 | 182 | 5,018,321 |
|  | Overlapping | 0 | 0 | 132 | 3,162,381 | 0 | 0 | 0 | 0 | 132 | 3,162,381 |
|  | Gap | 5 | 354,110 | 2 | 358,423 | 0 | 0 | 0 | 0 | 7 | 712,533 |
| Central Africa Republic | TOTAL | 0 | 0 | 8 | 541,289 | 0 | 0 | 8 | 398,465 | 16 | 939,754 |
|  | Treated LF | 0 | 0 | 4 | 85,166 | 0 | 0 | 0 | 0 | 4 | 85,166 |
|  | Treated STH | 0 | 0 | 0 | 0 | 0 | 0 | 0 | 0 | 0 | 0 |
|  | Overlapping | 0 | 0 | 0 | 0 | 0 | 0 | 0 | 0 | 0 | 0 |
|  | Gap | 0 | 0 | 4 | 456,123 | 0 | 0 | 8 | 398,465 | 12 | 854,588 |
| CONGO | TOTAL | 0 | 0 | 9 | 164,395 | 0 | 0 | 16 | 239,306 | 25 | 403,701 |
|  | Treated LF | 0 | 0 | 4 | 14,507 | 0 | 0 | 0 | 0 | 4 | 14,507 |
|  | Treated STH | 0 | 0 | 1 | 3,740 | 0 | 0 | 3 | 2,775 | 4 | 6,515 |
|  | Overlapping | 0 | 0 | 1 | 3,740 | 0 | 0 | 0 | 0 | 1 | 3,740 |
|  | Gap | 0 | 0 | 4 | 146,148 | 0 | 0 | 13 | 236,531 | 17 | 382,679 |
| COTE D'IVOIRE | TOTAL | 21 | 1,859,966 | 61 | 4,520,107 | 0 | 0 | 0 | 0 | 82 | 6,380,073 |
|  | Treated LF | 0 | 0 | 33 | 1,751,544 | 0 | 0 | 0 | 0 | 33 | 1,751,544 |
|  | Treated STH | 0 | 0 | 0 | 0 | 0 | 0 | 0 | 0 | 0 | 0 |
|  | Overlapping | 0 | 0 | 0 | 0 | 0 | 0 | 0 | 0 | 0 | 0 |
|  | Gap | 21 | 1,859,966 | 28 | 2,768,563 | 0 | 0 | 0 | 0 | 49 | 4,628,529 |
| Democratic Republic of Congo | TOTAL | 263 | 15,260,295 | 220 | 11,080,973 | 0 | 0 | 21 | 1,095,238 | 504 | 27,436,506 |
|  | Treated LF | 0 | 0 | 54 | 2,389,718 | 0 | 0 | 0 | 0 | 54 | 2,389,718 |
|  | Treated STH | 52 | 2,135,901 | 8 | 213,938 | 0 | 0 | 0 | 0 | 60 | 2,349,839 |
|  | Overlapping | 0 | 0 | 0 | 0 | 0 | 0 | 0 | 0 | 0 | 0 |
|  | Gap | 211 | 13,124,394 | 158 | 8,477,317 | 0 | 0 | 21 | 1,095,238 | 390 | 22,696,949 |
| ETHIOPIA | TOTAL | 699 | 19,174,338 | 110 | 2,689,434 | 0 | 0 | 0 | 0 | 809 | 21,863,772 |
|  | Treated LF | 0 | 0 | 33 | 464,010 | 0 | 0 | 0 | 0 | 33 | 464,010 |
|  | Treated STH | 212 | 6,514,295 | 44 | 1,343,902 | 0 | 0 | 0 | 0 | 256 | 7,858,197 |
|  | Overlapping | 0 | 0 | 11 | 243,004 | 0 | 0 | 0 | 0 | 11 | 243,004 |
|  | Gap | 487 | 12,660,043 | 44 | 1,124,526 | 0 | 0 | 0 | 0 | 531 | 13,784,569 |
| GHANA | TOTAL | 118 | 3,465,752 | 93 | 3,087,535 | 5 | 128,773 | 0 | 0 | 216 | 6,682,060 |
|  | Treated LF | 0 | 0 | 81 | 1,735,099 | 0 | 0 | 0 | 0 | 81 | 1,735,099 |
|  | Treated STH | 62 | 987,179 | 31 | 594,587 | 2 | 40,469 | 0 | 0 | 95 | 1,622,235 |
|  | Overlapping | 0 | 0 | 31 | 484,069 | 0 | 0 | 0 | 0 | 31 | 484,069 |
|  | Gap | 56 | 2,478,573 | 0 | 757,849 | 3 | 88,304 | 0 | 0 | 59 | 3,324,726 |
| GUINEA | TOTAL | 14 | 954,507 | 24 | 1,761,199 | 0 | 0 | 0 | 0 | 38 | 2,715,706 |
|  | Treated LF | 0 | 0 | 0 | 0 | 0 | 0 | 0 | 0 | 0 | 0 |
|  | Treated STH | 0 | 0 | 0 | 0 | 0 | 0 | 0 | 0 | 0 | 0 |
|  | Overlapping | 0 | 0 | 0 | 0 | 0 | 0 | 0 | 0 | 0 | 0 |
|  | Gap | 14 | 954,507 | 24 | 1,761,199 | 0 | 0 | 0 | 0 | 38 | 2,715,706 |
| GUINEE BISSAU | TOTAL | 0 | 0 | 33 | 128,428 | 0 | 0 | 0 | 0 | 33 | 128,428 |
|  | Treated LF | 0 | 0 | 33 | 94,821 | 0 | 0 | 0 | 0 | 33 | 94,821 |
|  | Treated STH | 0 | 0 | 0 | 0 | 0 | 0 | 0 | 0 | 0 | 0 |
|  | Overlapping | 0 | 0 | 0 | 0 | 0 | 0 | 0 | 0 | 0 | 0 |
|  | Gap | 0 | 0 | 0 | 33,607 | 0 | 0 | 0 | 0 | 0 | 33,607 |
| KENYA | TOTAL | 146 | 10,289,185 | 12 | 900,849 | 0 | 0 | 0 | 0 | 158 | 11,190,034 |
|  | Treated LF | 0 | 0 | 0 | 0 | 0 | 0 | 0 | 0 | 0 | 0 |
|  | Treated STH | 54 | 3,665,170 | 12 | 627,856 | 0 | 0 | 0 | 0 | 66 | 4,293,026 |
|  | Overlapping | 0 | 0 | 0 | 0 | 0 | 0 | 0 | 0 | 0 | 0 |
|  | Gap | 92 | 6,624,015 | 0 | 272,993 | 0 | 0 | 0 | 0 | 92 | 6,897,008 |
| MADAGASCAR | TOTAL | 14 | 1,296,358 | 98 | 5,046,066 | 0 | 0 | 0 | 0 | 112 | 6,342,424 |
|  | Treated LF | 0 | 0 | 61 | 2,415,662 | 0 | 0 | 0 | 0 | 61 | 2,415,662 |
|  | Treated STH | 5 | 341,993 | 82 | 4,185,032 | 0 | 0 | 0 | 0 | 87 | 4,527,025 |
|  | Overlapping | 0 | 0 | 57 | 2,256,184 | 0 | 0 | 0 | 0 | 57 | 2,256,184 |
|  | Gap | 9 | 954,365 | 12 | 701,556 | 0 | 0 | 0 | 0 | 21 | 1,655,921 |
| MALI | TOTAL | 0 | 0 | 61 | 4,956,727 | 2 | 210,785 | 0 | 0 | 63 | 5,167,512 |
|  | Treated LF | 0 | 0 | 13 | 593,397 | 0 | 0 | 0 | 0 | 13 | 593,397 |
|  | Treated STH | 0 | 0 | 0 | 0 | 0 | 0 | 0 | 0 | 0 | 0 |
|  | Overlapping | 0 | 0 | 0 | 0 | 0 | 0 | 0 | 0 | 0 | 0 |
|  | Gap | 0 | 0 | 48 | 4,363,330 | 2 | 210,785 | 0 | 0 | 50 | 4,574,115 |
| MOZAMBIQUE | TOTAL | 44 | 1,910,625 | 104 | 5,101,105 | 0 | 0 | 0 | 0 | 148 | 7,011,730 |
|  | Treated LF | 0 | 0 | 104 | 6,608,156 | 0 | 0 | 0 | 0 | 104 | 6,608,156 |
|  | Treated STH | 41 | 1,686,325 | 38 | 1,834,590 | 0 | 0 | 0 | 0 | 79 | 3,520,915 |
|  | Overlapping | 0 | 0 | 38 | 1,701,644 | 0 | 0 | 0 | 0 | 38 | 1,701,644 |
|  | Gap | 3 | 224,300 | 0 | 0 | 0 | 0 | 0 | 0 | 3 | 224,300 |
| NIGER | TOTAL | 9 | 741,570 | 28 | 3,071,035 | 0 | 0 | 0 | 0 | 37 | 3,812,605 |
|  | Treated LF | 0 | 0 | 28 | 2,739,896 | 0 | 0 | 0 | 0 | 28 | 2,739,896 |
|  | Treated STH | 6 | 608,870 | 0 | 0 | 0 | 0 | 0 | 0 | 6 | 608,870 |
|  | Overlapping | 0 | 0 | 0 | 0 | 0 | 0 | 0 | 0 | 0 | 0 |
|  | Gap | 3 | 132,700 | 0 | 331,139 | 0 | 0 | 0 | 0 | 3 | 463,839 |
| NIGERIA | TOTAL | 185 | 12,461,108 | 545 | 32,773,088 | 29 | 1,679,581 | 13 | 725,031 | 772 | 47,638,808 |
|  | Treated LF | 4 | 109,952 | 348 | 14,987,715 | 0 | 0 | 0 | 0 | 352 | 15,097,667 |
|  | Treated STH | 28 | 844,580 | 118 | 4,515,836 | 29 | 1,681,684 | 0 | 0 | 175 | 7,042,100 |
|  | Overlapping | 2 | 55,207 | 101 | 2,648,862 | 0 | 0 | 0 | 0 | 103 | 2,704,069 |
|  | Gap | 155 | 11,561,783 | 180 | 15,918,399 | 0 | 0 | 13 | 725,031 | 348 | 28,205,213 |
| SENEGAL | TOTAL | 26 | 1,649,682 | 50 | 2,407,680 | 0 | 0 | 0 | 0 | 76 | 4,057,362 |
|  | Treated LF | 0 | 0 | 6 | 374,730 | 0 | 0 | 0 | 0 | 6 | 374,730 |
|  | Treated STH | 26 | 1,594,018 | 44 | 1,603,996 | 0 | 0 | 0 | 0 | 70 | 3,198,014 |
|  | Overlapping | 0 | 0 | 0 | 0 | 0 | 0 | 0 | 0 | 0 | 0 |
|  | Gap | 0 | 55,664 | 0 | 428,954 | 0 | 0 | 0 | 0 | 0 | 484,618 |
| TANZANIA | TOTAL | 63 | 3,479,472 | 107 | 5,461,777 | 6 | 268,202 | 0 | 0 | 166 | 9,209,451 |
|  | Treated LF | 0 | 0 | 105 | 5,681,849 | 5 | 212,116 | 0 | 0 | 100 | 5,893,965 |
|  | Treated STH | 0 | 0 | 61 | 2,547,967 | 2 | 113,436 | 0 | 0 | 63 | 2,661,403 |
|  | Overlapping | 0 | 0 | 61 | 2,154,393 | 2 | 81,378 | 0 | 0 | 63 | 2,235,771 |
|  | Gap | 63 | 3,479,472 | 2 | 0 | 1 | 24,028 | 0 | 0 | 66 | 3,503,500 |
| UGANDA | TOTAL | 58 | 6,577,037 | 54 | 4,429,028 | 0 | 0 | 0 | 0 | 112 | 11,006,065 |
|  | Treated LF | 0 | 0 | 48 | 3,054,621 | 0 | 0 | 0 | 0 | 48 | 3,054,621 |
|  | Treated STH | 0 | 0 | 2 | 1,186 | 0 | 0 | 0 | 0 | 2 | 1,186 |
|  | Overlapping | 0 | 0 | 2 | 1,186 | 0 | 0 | 0 | 0 | 2 | 1,186 |
|  | Gap | 58 | 6,577,037 | 6 | 1,374,407 | 0 | 0 | 0 | 0 | 64 | 7,951,444 |
| **AMRO** |  |  |  |  |  |  |  |  |  |  |  |
| Brazil | TOTAL | 0 | 0 | 2 | 19,050 | 27 | 8,456,269 | 0 | 0 | 29 | 8,475,319 |
|  | Treated LF | 0 | 0 | 2 | 19,050 | 0 | 0 | 0 | 0 | 2 | 19,050 |
|  | Treated STH | 0 | 0 | 0 | 0 | 27 | 4,754,092 | 0 | 0 | 27 | 4,754,092 |
|  | Overlapping | 0 | 0 | 0 | 0 | 0 | 0 | 0 | 0 | 0 | 0 |
|  | Gap | 0 | 0 | 0 | 0 | 0 | 27,753,562 |  |  | 0 | 27,753,562 |
| HAITI | TOTAL | 0 | 0 | 106 | 2,308,189 | 34 | 342,936 | 0 | 0 | 140 | 2,651,125 |
|  | Treated LF | 0 | 0 | 77 | 1,276,470 | 0 | 0 | 0 | 0 | 77 | 1,276,470 |
|  | Treated STH | 0 | 0 | 6 | 604,000 | 0 | 0 | 0 | 0 | 6 | 604,000 |
|  | Overlapping | 0 | 0 | 3 | 103,567 | 0 | 0 | 0 | 0 | 3 | 103,567 |
|  | Gap | 0 | 0 | 23 | 427,719 | 34 | 342,936 | 0 | 0 | 57 | 770,655 |
| GUYANA | TOTAL | 0 | 0 | 10 | 178,986 | 0 | 0 | 0 | 0 | 10 | 178,986 |
|  | Treated LF | 0 | 0 | 2 | 49,317 | 0 | 0 | 0 | 0 | 2 | 49,317 |
|  | Treated STH | 0 | 0 | 0 | 0 | 0 | 0 | 0 | 0 | 0 | 0 |
|  | Overlapping | 0 | 0 | 0 | 0 | 0 | 0 | 0 | 0 | 0 | 0 |
|  | Gap | 0 | 0 | 8 | 129,669 | 0 | 0 | 0 | 0 | 8 | 129,669 |
| DOMINICAN REPUBLIC | TOTAL | 22 | 1,574,968 | 5 | 217,763 | 5 | 322,363 | 0 | 0 | 32 | 2,115,094 |
|  | Treated LF | 0 | 0 | 0 | 0 | 0 | 0 | 0 | 0 | 0 | 0 |
|  | Treated STH | 22 | 1,172,786 | 5 | 155,163 | 5 | 180,774 | 0 | 0 | 32 | 1,508,723 |
|  | Overlapping | 0 | 0 | 0 | 0 | 0 | 0 | 0 | 0 | 0 | 0 |
|  | Gap | 0 | 402,182 | 0 | 62,600 | 0 | 141,589 | 0 | 0 | 0 | 606,371 |
| **EMRO** |  |  |  |  |  |  |  |  |  |  |  |
| SUDAN | TOTAL | 24 | 1,279,742 | 30 | 1,694,741 | 0 | 0 | 130 | 8,126,024 | 184 | 11,100,507 |
|  | Treated LF | 0 | 0 | 0 | 0 | 0 | 0 | 0 | 0 | 0 | 0 |
|  | Treated STH | 2 | 5,533 | 0 | 0 | 0 | 0 | 12 | 325,493 | 14 | 331,026 |
|  | Overlapping | 0 | 0 | 0 | 0 | 0 | 0 | 0 | 0 | 0 | 0 |
|  | Gap | 22 | 1,274,209 | 30 | 1,694,741 | 0 | 0 | 118 | 7,800,531 | 170 | 10,769,481 |
| **SEARO** |  |  |  |  |  |  |  |  |  |  |  |
| BANGLADESH | TOTAL | 35 | 19,762,339 | 2 | 955,124 | 18 | 6,757,629 | 0 | 0 | 55 | 27,475,092 |
|  | Treated LF | 0 | 0 | 0 | 0 | 0 | 0 | 0 | 0 | 0 | 0 |
|  | Treated STH | 35 | 17,017,799 | 2 | 1,627,545 | 18 | 5,708,316 | 0 | 0 | 55 | 24,353,660 |
|  | Overlapping | 0 | 0 | 0 | 0 | 0 | 0 | 0 | 0 | 0 | 0 |
|  | Gap | 0 | 2,744,540 | 0 | 0 | 0 | 1,049,313 | 0 | 0 | 0 | 3,793,853 |
| INDIA | TOTAL | 0 | 0 | 184 | 103,383,636 | 71 | 33,183,326 | 0 | 0 | 255 | 136,566,962 |
|  | Treated LF | 0 | 0 | 169 | 69,402,615 | 0 | 0 | 0 | 0 | 169 | 69,402,615 |
|  | Treated STH | 0 | 0 | 36 | 14,895,184 | 0 | 0 | 0 | 0 | 36 | 14,895,184 |
|  | Overlapping | 0 | 0 | 23 | 8,033,599 | 0 | 0 | 0 | 0 | 23 | 8,033,599 |
|  | Gap | 0 | 0 | 74 | 27,119,436 | 71 | 33,183,326 | 0 | 0 | 145 | 60,302,762 |
| INDONESIA | TOTAL | 269 | 23,075,092 | 205 | 13,528,884 | 36 | 2,110,779 | 1 | 326,310 | 511 | 39,041,065 |
|  | Treated LF | 0 | 0 | 87 | 4,285,049 | 0 | 0 | 0 | 0 | 87 | 4,285,049 |
|  | Treated STH | 0 | 0 | 0 | 0 | 0 | 0 | 0 | 0 | 0 | 0 |
|  | Overlapping | 0 | 0 | 0 | 0 | 0 | 0 | 0 | 0 | 0 | 0 |
|  | Gap | 269 | 23,075,092 | 118 | 9,243,835 | 36 | 2,110,779 | 1 | 326,310 | 424 | 34,756,016 |
| MYANMAR | TOTAL | 20 | 1,070,908 | 42 | 6,415,229 | 7 | 788,087 | 0 | 0 | 69 | 8,274,224 |
|  | Treated LF | 0 | 0 | 39 | 5,455,811 | 0 | 0 | 0 | 0 | 39 | 5,455,811 |
|  | Treated STH | 20 | 1,032,808 | 42 | 6,171,505 | 7 | 681,539 | 0 | 0 | 69 | 7,885,852 |
|  | Overlapping | 0 | 0 | 39 | 5,438,661 | 0 | 0 | 0 | 0 | 39 | 5,438,661 |
|  | Gap | 0 | 38,100 | 0 | 243,724 | 0 | 106,548 | 0 | 0 | 0 | 388,372 |
| NEPAL | TOTAL | 14 | 425,109 | 41 | 4,384,417 | 20 | 2,716,532 | 0 | 0 | 75 | 7,526,058 |
|  | Treated LF | 0 | 0 | 41 | 3,019,859 | 0 | 0 | 0 | 0 | 41 | 3,019,859 |
|  | Treated STH | 14 | 389,038 | 36 | 558,035 | 18 | 1,075,919 | 0 | 0 | 68 | 2,022,992 |
|  | Overlapping | 0 | 0 | 36 | 533,823 | 0 | 0 | 0 | 0 | 36 | 533,823 |
|  | Gap | 0 | 36,071 | 0 | 806,523 | 2 | 1,640,613 | 0 | 0 | 2 | 2,483,207 |
| **WPRO** |  |  |  |  |  |  |  |  |  |  |  |
| PHILIPPINES | TOTAL | 36 | 11,142,460 | 22 | 4,218,431 | 23 | 3,894,861 | 0 | 0 | 81 | 19,255,752 |
|  | Treated LF | 0 | 0 | 22 | 4,218,431 | 0 | 0 | 0 | 0 | 22 | 4,218,431 |
|  | Treated STH | 27 | 4,571,347 | 17 | 1,980,755 | 18 | 1,579,257 | 0 | 0 | 62 | 8,131,359 |
|  | Overlapping | 0 | 0 | 17 | 1,909,761 | 0 | 0 | 0 | 0 | 17 | 1,909,761 |
|  | Gap | 9 | 6,571,113 | 0 | 0 | 5 | 2,315,604 | 0 | 0 | 14 | 8,886,717 |

*SAC population includes districts with STH prevalence below 20%

(Source of data: PCT databank)
